# Cisplatin Binding to Human Serum Transferrin: A Crystallographic Study

**DOI:** 10.1021/acs.inorgchem.2c04206

**Published:** 2023-01-05

**Authors:** Romualdo Troisi, Francesco Galardo, Giarita Ferraro, Filomena Sica, Antonello Merlino

**Affiliations:** † Department of Chemical Sciences, University of Naples Federico II, Complesso Universitario di Monte Sant’Angelo, via Cintia, Naples I-80126, Italy

## Abstract

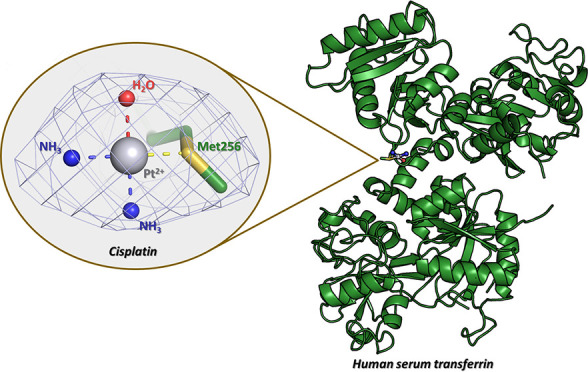

The molecular mechanism of how human serum transferrin (hTF) recognizes cisplatin at the atomic level is still unclear. Here, we report the molecular structure of the adduct formed upon the reaction of hTF with cisplatin. Pt binds the side chain of Met256 (at the N-lobe), without altering the protein overall conformation.

## Abstract 2

The molecular structure of the adduct formed upon the reaction of cisplatin with human serum transferrin (hTF) with Fe^3+^ bound at the C-lobe (Fe_C_-hTF) was solved. Cisplatin binding does not significantly alter the overall conformation of Fe_C_-hTF. The metal compound binds the protein close to the side chain of Met256 at the N-lobe.

Cisplatin, *cis*-diammineplatinum(II) dichloride, is a DNA-damaging anticancer agent widely used for the treatment of many forms of solid tumors.^1−6^ It works by interfering with DNA replication and transcription as a result of the creation of intrastrand cross-linked DNA adducts, which ultimately results in the death of cancer cells.^7−11^ Cisplatin also exhibits serious side effects that are possibly related to enzymatic and protein structural changes,^12^ frequently restricting its therapeutic uses.

Although DNA is the primary biological target of cisplatin, the interactions of this metallodrug with other biological macromolecules are of great interest because they are crucial in regulating drug biodistribution, efficacy, and toxicity.^13−16^

Human serum transferrin (hTF) is abundant in the plasma with an average blood content of 200–370 mg/dL in healthy people. It binds Fe^3+^ and delivers it to cells through the transferrin receptor (TFR).

hTF is a ∼80 kDa single-chain protein consisting of two lobes (called the N- and C-lobes), each comprising almost 330 residues, separated by a short flexible linker (residues 331–339).^17^ Each lobe can be further divided into two similar domains: N1 (residues 1–92 and 247–330), N2 (residues 93–246), C1 (residues 320–425 and 573–679), and C2 (residues 426–572). Both the N and C domains are separated by a cleft, where a Fe binding site is located. Remarkably, upon Fe^3+^ binding, the domains of each lobe rotate relative to one another, thereby reducing the solvent accessibility of the two equivalent Fe binding sites.^18^ Thus, the apo conformation is described as “open”, while the Fe-bound form is denoted as “closed”.

Because TFR is overexpressed on cancer cells,^19^ hTF has been proposed as a potential anticancer drug carrier.^20^ In this frame, it has been demonstrated that hTF can bind cisplatin and selectively deliver it to cancer cells *in vitro* and *in vivo*.^21,22^ Obviously, the binding of cisplatin to the protein can also potentially impact its efficacy as an anticancer agent.

Although numerous studies have been carried out to establish the exact molecular mechanism of how hTF binds cisplatin,^22−29^ controversial opinions still exist on cisplatin binding sites of hTF. Early studies by Elliott et al. reported binding of one or two cisplatin fragments per hTF molecule.^23^ Conversely, in 1995, Hoshino et al. suggested that, in contrast to Fe ions, cisplatin binds hTF at a single Pt binding site.^24^ A few years later, using NMR spectroscopy data, Sadler and co-workers suggested that cisplatin binding to hTF involves the side chain of Met256. This conclusion was drawn from the observation of a substantial chemical shift change of the ^13^C-methyl-Met256 resonance when the protein is treated with cisplatin, which is not observed when hTF is incubated with Fe salts.^25^ Subsequent mass spectrometry, UV–vis absorption spectroscopy, and molecular modeling experiments by Allardyce, Dyson, and co-workers suggested that the hydroxy group of Thr457 is the most likely Pt binding site of hTF.^26,27^ Note that Thr457 is located close to the Fe^3+^ binding site on the C-lobe of the protein. Further experiments using hyphenated multidimensional liquid chromatography and electrospray ionization tandem mass spectrometry highlighted a variety of cisplatin binding sites close to Met256, Glu265, Tyr314, Glu385, and Thr457.^28^ In 2012, Luo et al. found that hTF can bind more than 22 cisplatin fragments, and the adduct formed upon reaction of the Pt-based drug with the protein can specifically deliver cisplatin to human hepatocellular liver carcinoma cell lines, facilitating apoptosis via a mechanism that is distinct from that of free cisplatin.^22^ Recently, it has been shown that when hTF is pretreated with 10% ethanol, the number of cisplatin binding sites for a protein molecule could increase to 55, remaining stable at 41 for at least 1 week.^29^

Thus, from a survey of this literature data, it appears clear that the cisplatin binding sites of hTF have not yet been unambiguously identified, mainly because of a substantial lack of direct structural information on the cisplatin/hTF system.

Here, we report for the first time the result of the X-ray structure determination of the adduct formed upon reaction of the Pt drug with hTF. We use the hTF form with Fe^3+^ bound at the C-lobe only (Fe_C_-hTF) because crystals of this form have already been used to obtain adducts of hFT with metal ions.^30−33^ Moreover, Fe_C_-hTF represents a large fraction of hTF species in serum.^18,33−35^

Crystals of the cisplatin/Fe_C_-hTF adduct were thus obtained by using the soaking strategy.^31,33^ In particular, crystals of Fe_C_-hTF were grown by a hanging-drop vapor diffusion method at 20 °C using a reservoir solution consisting of 15% (w/v) PEG 3350, 16% (v/v) glycerol, 8 mM disodium malonate, and 150 mM Na-PIPES (pH 6.5). These crystals were then soaked for 72 h in a cryoprotectant solution saturated with cisplatin (see the Experimental Section for further details). X-ray diffraction data were collected on these crystals at 100 K on the XRD2 beamline of Elettra Sincrotrone Trieste, Italy (see Table S1 for data collection statistics). Crystals belong to the space group *C*222_1_, diffract X-ray at 3.17 Å resolution, and present a single hTF polypeptide chain in the asymmetric unit. The structure was solved by the molecular replacement method using the program *Phaser MR*(36,37) and the coordinates of Fe_C_-hTF from the Protein Data Bank (PDB) code 4X1B,^30^ stripped of all its ligands, as the search model. The final model (Figure 1), which includes some regions in the C-lobe that are absent in the starting model (for example, residues 418–423 and 612–623, which are very flexible, and Asn413 N-glycosylation), was refined using the *REFMAC5*(37,38) program to an R-factor of 0.177 (R-free = 0.243) with good stereochemistry (see Table S1 for refinement statistics). Deviations from ideal bond lengths and angles are 0.001 Å and 0.98°, respectively. Notably, the overall conformation of the protein is not significantly affected by cisplatin binding (Figure S1): the Cα root-mean-square deviation of the cisplatin/Fe_C_-hTF adduct from the starting model and from other reported^30−33,39^ structures of M_C_-hTF (M = metal) is 0.39 Å and within the range of 0.40–0.53 Å, respectively. Accordingly, there are no major changes in the orientation of the two lobes and of their domains when cisplatin binds the protein (Figures 1 and S1): the C-lobe adopts a closed conformation, whereas the N-lobe adopts an open conformation.

**Figure 1:**
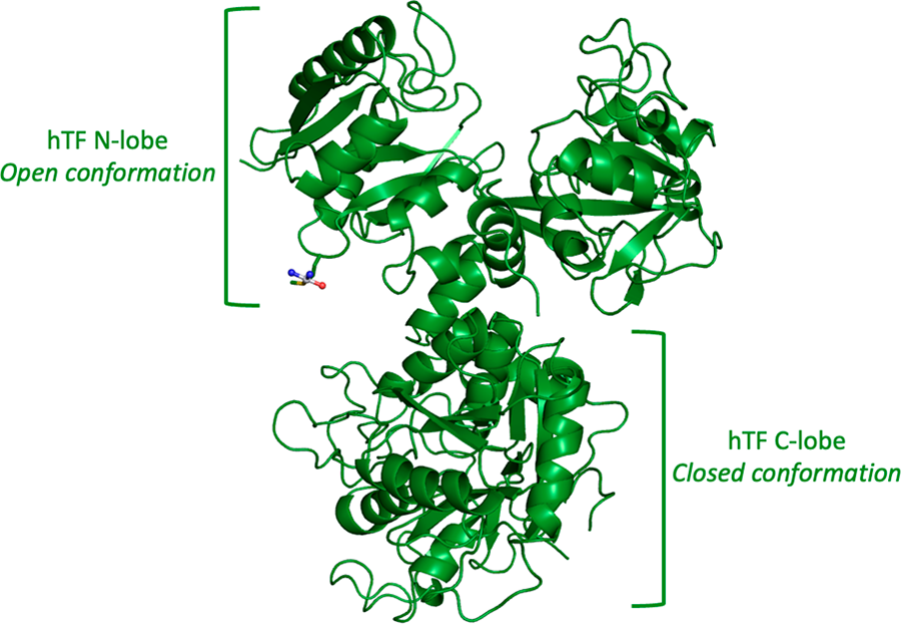
Overall structure of the cisplatin/Fe_C_-hTF adduct. A single cisplatin fragment has been identified close to the side chain of Met256 in the N-lobe. The cisplatin fragment atoms are shown as spheres (Pt is gray, NH_3_ are blue, and H_2_O is red), and the residue that coordinates the Pt center is reported as a stick. Coordinates and structure factors of the cisplatin/Fe_C_-hTF adduct were deposited in the PDB under the accession code 8BRC.

Inspection of the difference Fourier (2*F*_o_ – *F*_c_ and *F*_o_ – *F*_c_) and anomalous difference electron density maps clearly revealed the presence of a peak in correspondence with the Fe binding site at the C-lobe, close to residues Asp392, Tyr426, Tyr517, and His585 (Figure 2A) and of a peak close to the side chain of Met256 at the N-lobe (Figure 2B). Close to the Fe (anomalous peak at 5.20σ), the synergistic anion malonate, present in the crystallization condition, was added to the model, as was done in the starting model and in other M_C_-hTF structures.^30−33,39^ The peak close to Met256 has been attributed to a Pt center. Here, an anomalous peak is at 5.22σ. A comparison between the 2*F*_o_ – *F*_c_ electron density map of Met256 in our structure and in the other structure^30−33,39−46^ of hTF deposited in the PDB is reported in Figure S2. At the Pt binding site, the Pt ligands have been tentatively assigned, but because of the limited resolution of the structure, the ligand assignments should be considered with care. In particular, considering the experimental conditions (pH 6.5 and the absence of chloride ions), the long soaking time (72 h), and the absence of an anomalous difference electron density map peak in correspondence with the Pt ligands, in addition to Met256, two NH_3_ groups and one H_2_O molecule have been assigned as Pt ligands (Figure 2B).

**Figure 2:**
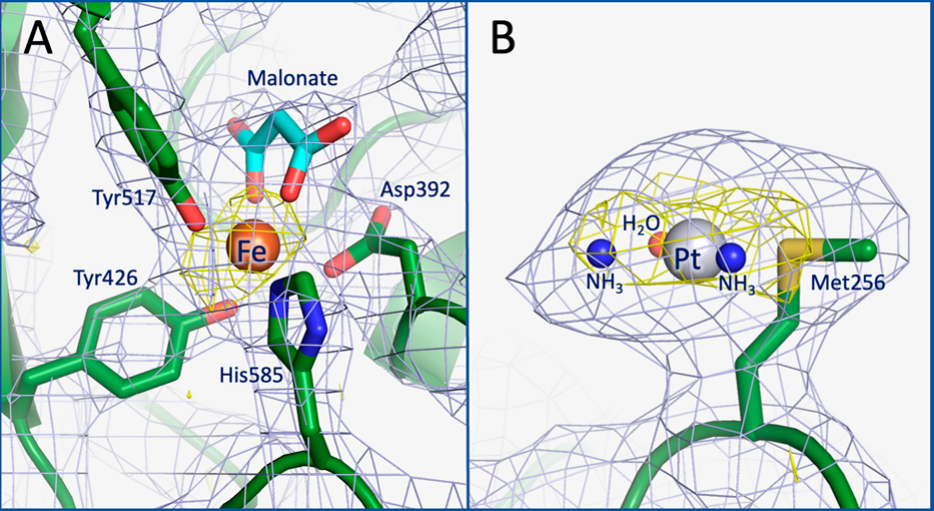
Details of the binding sites of Fe ion (A) and cisplatin (B) in the structure of the cisplatin/Fe_C_-hTF adduct. 2*F*_o_ – *F*_c_ electron density maps (gray) are contoured at the 1.0σ level, and anomalous difference electron density maps are in yellow.

The cisplatin binding site is located on the protein surface at ∼35 Å from the Fe^3+^ ion in the C-lobe and at ∼30 Å from the Fe binding site in the N-lobe. Refinements indicate an occupancy value of ∼0.6 for the Pt ion and of 1.0 for the Fe ion. *B*-factors for the metal centers are high but with values not far from those of the coordinating residues (*B*-factor ratios within the range 0.8–1.4). The average Pt···Sδ(Met256) distance is 2.2 Å, in line with the expectation.^47^

Attempts to improve the resolution of the structure of the cisplatin/Fe_C_-hTF adduct carried out to date failed. However, to obtain further evidence of the Pt binding site, anomalous difference electron density maps have been recalculated at lower resolution, where the *I*/σ ratio is higher using the data set at 3.17 Å resolution and analyzing the additional X-ray diffraction data collected on other cisplatin/Fe_C_-hTF adduct crystals at a similar or lower resolution (data set 2 at 3.22 Å resolution and data set 3 at 3.63 Å resolution, respectively). These data have also been compared with those derived from a data set (at 4.02 Å resolution) collected on a Pt-free Fe_C_-hTF (Table S2). Only an anomalous peak at 4.54σ in correspondence with Fe^3+^ in the C-lobe was observed in the case of the Pt-free protein structure, while significant anomalous peaks have been observed close to Met256 in the Pt-bound structures. Finally, the reaction of cisplatin with Sδ of Met256 has been further highlighted by the omit *F*_o_ – *F*_c_ electron density map obtained by removing the Met256 side chain and the coordinating compound from the structure of the cisplatin/Fe_C_-hTF adduct (Figure S3).

In conclusion, we have solved and refined, for the first time, the 3D structure of an adduct formed in the reaction of cisplatin with hTF. The main results of this study can be summarized as follows:

(i) The first direct information on the location of a binding site for cisplatin on the hTF structure has been reported. Cisplatin binds Fe_C_-hTF close to the side chain of Met256 at the N-lobe. This result is in line with that obtained in other cisplatin/protein adducts, which indicated that cisplatin binding to proteins occurs mainly at the level of the side chains of His or Met residues^47−51^ and with early NMR spectroscopy and mass spectrometry studies by Sadler and co-workers^25^ and by Will, Wolters, and Sheldrick.^28^

(ii) Cisplatin binding to hTF does not significantly alter the overall conformation of the protein. In the platinated Fe_C_-hTF, the C-lobe is in a closed conformation, whereas the N-lobe adopts an open state.

(iii) The cisplatin binding site is distinct from those previously found for Ru^3+^ and Os^3+^ (His14/His289, His273, His349/His350, Lys489, Lys490/Glu507, and His578/Arg581),^33^ for Fe^3+^ (Fe binding site),^30,33,39,41,42,44,45^ Ti^4+^ (Fe binding site of the C-lobe, Tyr188),^31,33,43^ Yb^3+^ (Fe binding site of the C-lobe),^30^ Cr^3+^ (Fe binding site of the C-lobe),^32^ and Bi^3+^ (Tyr188)^41^ (Table S3). This finding indicates that, in principle, it is possible to design anticancer metal-based drugs/hTF adducts where the protein can carry cisplatin and other anticancer metallodrugs. In this respect, it is interesting to note that, although solved at a relatively low resolution, the crystal structure of the cisplatin/Fe_C_-hTF adduct here reported (PDB code:8BRC) can serve as an excellent template for the design of new theranostic agents, given the ability of hTF to transport both anticancer agents, like cisplatin, and radio-imaging agents.^35^

Collectively, this work does not solve the literature debates on the number and location of Pt binding sites on the hTF structure, but for sure it provides solid evidence that the side chain of Met256 is involved in the cisplatin recognition.

As a final note, it is useful to underline that our structure enriches the repertoire of structures of hTF adducts with metal compounds that is still scarce (Table S3) and provides critical data for our understanding of the role of hTF in cisplatin cellular delivery and for interpreting the results of physicochemical experiments carried out so far on this system.

## Supporting Information Available

Experimental section (materials, crystallization, and data collection, structure determination, refinement, and structural analysis), supplemental tables (Tables S1–S3), and supplemental figures (Figures S1–S3) (PDF)
