# Supplementary material for: Cisplatin Binding to Human Serum Transferrin: A Crystallographic Study
Source: Inorg Chem. 2023 Jan 5;62(2):675–8. doi: 10.1021/acs.inorgchem.2c04206 (PMC9846693; doi:10.1021/acs.inorgchem.2c04206)
Supplement: Supplementary file 1 — ic2c04206_si_001.pdf [file ic2c04206_si_001.pdf]

Supporting Information

Cisplatin binding to human serum transferrin: a crystallographic study

Romualdo Troisi,[a] Francesco Galardo,[a] Giarita Ferraro,[a] Filomena Sica[a] and Antonello Merlino[a,]*

[a] Department of Chemical Sciences, University of Naples Federico II, Complesso Universitario di Monte Sant'Angelo, via Cintia, I-80126, Naples, Italy.

* E-mail: antonello.merlino@unina.it

Experimental section

Materials

cis-Diammineplatinum(II) dichloride (cisplatin), human serum apo-transferrin (apo-hTF, T4382-100MG, in lyophilised form), and all chemicals were purchased from Sigma-Aldrich (Merck).

Crystallization

The 1:1 complex between hTF and Fe^{3+} (Fe_C-hTF) was prepared following a literature protocol,[1,2] with minor modifications. In detail, equimolar amounts of $NH_4Fe(SO_4)_2$ and apo-hTF were mixed in a solution containing 10 mM Na-HEPES pH 7.5 and 10 mM $NaHCO_3$, and incubated at 4 °C overnight. The Fe_C-hTF complex was concentrated to about 1 mM using a 10 kDa-cutoff Centricon mini-concentrator (Vivaspin 500, Sartorius) and a refrigerated centrifuge (Microfuge 20R - Beckman Coulter).

Fe_C-hTF crystals were grown in 15% (w/v) PEG 3350, 16% (v/v) glycerol, 8 mM disodium malonate, and 150 mM Na-PIPES pH 6.5 by hanging drop vapour diffusion method mixing 1 µL protein solution with 1 µL reservoir solution at 20 °C. Crystals of Fe_C-hTF were soaked for 72 h in a cryo-protectant solution [27% (w/v) PEG 3350, 30% (v/v) glycerol, 8 mM disodium malonate, and 50 mM Na-PIPES pH 6.5] saturated with cisplatin.

Data collection, structure determination, refinement, and structural analysis

Diffraction data were collected at the XRD2 beamline of Elettra Sincrotrone Trieste (Italy), using λ = 1.0000 Å. Datasets were processed using autoPROC software.[3-6] The phase problem was solved by molecular replacement using Phaser MR[5,7] and the coordinates of the native protein (PDB code: 4X1B),[1] as a search model. Restrained refinements were carried out using REFMAC5.[5,8] Coot program[9] was used for the visualization of the electron density maps and for model building. Pt binding site was identified using difference Fourier ($2F_o$-F_c and F_o-F_c) and anomalous difference electron density maps. Pt occupancy was evaluated trying to minimize the positive and negative peaks on metal centres in the Fourier difference F_o–F_c electron density maps and to obtain the best R_{factor} and R_{free} values. Structures were validated using the PDB validation server (https://validate-rcsb-1.wwpdb.org/) and Coot routines.[9] Root-mean-square deviations (RMSD) were calculated using the Superpose program (CCP4 package).[5] The coordinates of the cisplatin/Fe_C-hTF adduct structure obtained at highest resolution (dataset 1) were deposited in the Protein Data Bank (PDB code: 8BRC). Detailed statistics on the data collection and refinement are reported in Table S1. Molecular graphics figures were prepared with PyMOL (DeLano Scientific, Palo Alto, CA, USA).

Supplemental tables

Table S1. Data collection and refinement statistics. Values in brackets refer to the highest resolution shell.

	**cisplatin/Fe_C-hTF adduct (dataset 1)**
Crystal data	
**Space group**	$C222_1$
**Unit-cell parameters**	
**a, b, c (Å)**	136.34, 156.40, 107.44
**α, β, γ (°)**	90.00, 90.00, 90.00
**No. of molecules in the asymmetric unit**	1
Data collection	
**Resolution limits (Å)**	102.77 – 3.17 (3.22 – 3.17)
**No. of observations**	255200 (13121)
**No. of unique reflections**	19869 (969)
**Completeness (%)**	100.0 (100.0)
**$\langle I/\sigma(I)\rangle$**	13.7 (2.3)
**Average multiplicity**	12.8 (13.5)
**$CC_{1/2}$**	1.0 (0.8)
Refinement	
**Resolution limits (Å)**	102.77 – 3.17
**No. of reflections**	18841
**R_{factor}/R_{free}**	0.177/0.243
**No. of atoms**	5264
**Mean B value (Å^2)**	112.8
**RMSD from ideal values**	
**Bond lengths (Å)**	0.001
**Bond angles (°)**	0.979
**Ramachandran plot, residues in (%)**	
**Most favoured region**	91.7
**Additionally allowed region**	8.3
**Generously allowed region**	0
**Fe occupancy**	1.00
**Fe B-factors (Å^2)**	106.8
**Pt occupancy**	0.60
**Pt B-factors (Å^2)**	234.8
PDB code	8BRC

Table S2. Anomalous signal, calculated at different resolutions, close to Fe and Pt in the structures of Pt-free Fe_C-hTF and of cisplatin/Fe_C-hTF adducts from different datasets, derived from different crystals.

Structure	Dataset	Resolution	Fe	Pt
Cisplatin/Fe_C-hTF adduct	1	3.17 Å	5.20 σ	5.22 σ
		4.00 Å	7.98 σ	7.02 σ
		5.00 Å	7.61 σ	8.44 σ
	2	3.22 Å	6.45 σ	3.78 σ
		4.00 Å	7.44 σ	4.77 σ
		5.00 Å	8.39 σ	5.69 σ
	3	3.63 Å	< 3.5 σ	< 3.5 σ
		4.00 Å	< 3.5 σ	3.84 σ
		5.00 Å	< 3.5 σ	5.03 σ
Pt-free Fe_C-hTF	1	4.02 Å	4.54 σ	-
		5.00 Å	4.13 σ	-

Table S3. Structures of human transferrin (UniProtKB code: P02787) deposited in the PDB (October 2022). Only the structures of the full-length protein have been considered.

PDB code	Title	Resolution (Å)	Metal ions/metal compounds	Metal binding site	Other ligands	Transferrin form [a]	Reference	Notes
2HAU	Apo-Human Serum Transferrin (Non-Glycosylated)	2.70	-	-	-	Apo-hTF	10	2 molecules, containing Se-Met, in the asymmetric unit
2HAV	Apo-Human Serum Transferrin (Glycosylated)	2.70	-	-	-	Apo-hTF		2 molecules in the asymmetric unit
3QYT	Diferric bound human serum transferrin	2.80	Fe^{3+}	Asp392, Tyr426, Tyr517, His585	Carbonate ion	Fe_C-hTF	11	The second Fe^{3+} ion is bound to only two residues of the Fe biding site of N-lobe
			Fe^{3+}	Tyr95, Tyr188	Carbonate ion, sulphate ion			
4H0W	Bismuth bound human serum transferrin	2.40	Fe^{3+}	Asp392, Tyr426, Tyr517, His585	Carbonate ion	Fe_C-hTF		The Bi^{3+} ion is bound to only one residue of the Fe biding site of N-lobe
			Bi^{3+}	Tyr188	Carbonate ion, nitrilotriacetic acid			
3V83	The 2.1 angstrom crystal structure of diferric human transferrin	2.10	Fe^{3+}	A, B, D, E, F molecules: Asp392, Tyr426, Tyr517, His585<br><br>C molecule: Tyr426, Tyr517, His585	Bicarbonate ion	Fe_NFe_C-hTF	12	6 molecules in the asymmetric unit
			Fe^{3+}	Asp63, Tyr95, Tyr188, His249	Bicarbonate ion			
3V8X	The crystal structure of transferrin binding protein A (TbpA) from Neisserial meningitidis serogroup B in complex with full length human transferrin	2.60	-	-	-	Apo-hTF		Human transferrin in complex with transferrin binding protein A
4X1B	Human serum transferrin with ferric ion bound at the C-lobe only	2.45	Fe^{3+}	Asp392, Tyr426, Tyr517, His585	Malonate ion	Fe_C-hTF	1	-
4X1D	Ytterbium-bound human serum transferrin	2.80	Yb^{3+}	Asp392, Tyr426, Tyr517, His585	Malonate ion	Yb_C-hTF		2 molecules in the asymmetric unit
5DYH	Ti(IV) bound human serum transferrin	2.68	Ti^{4+}	Tyr426, Tyr517	Carbonate ion, citric acid	Apo-hTF	13	In the 2 molecules of the asymmetric unit, the Ti^{4+} ion is bound to only two residues of the Fe biding site of C-lobe

5H52	Structure of Titanium-bound human serum transferrin	3.00	Ti^{4+}	Asp392, Tyr426, Tyr517, His585	Malonate ion	Ti_C-hTF	14	The second Ti^{4+} ion is bound to only one residue of the Fe biding site of N-lobe
			Ti^{4+}	Tyr188	Citric acid, water			
5Y6K	Human serum transferrin bound to a fluorescent probe	2.86	Fe^{3+}	Asp392, Tyr426, Tyr517, His585	Malonate ion	Fe_C-hTF	15	The second Fe^{3+} ion is bound to only one residue of the Fe biding site of N-lobe
			Fe^{3+}	Tyr188	TRACER (fluorescent probe)			
6CTC	Crystal structure of human transferrin bound to Triferic FPC iron pyrophosphate	2.60	Fe^{3+}	Asp392, Tyr426, Tyr517, His585	Carbonate ion	Fe_C-hTF	16	The second Fe^{3+} ion is bound to only one residue of the Fe biding site of N-lobe
			Fe^{3+}	Tyr188	Pyrophosphate ion			
6D03	Cryo-EM structure of a Plasmodium vivax invasion complex essential for entry into human reticulocytes; one molecule of parasite ligand	3.68	Fe^{3+}	C molecule: Tyr426, Tyr517, His585<br><br>D molecule: Tyr426, Tyr517, His585, Arg632	Carbonate ion	Fe_NFe_C-hTF		Cryo-EM structure containing 2 molecules of human transferrin in complex with transferrin receptor protein and reticulocyte binding protein
			Fe^{3+}	C molecule: Tyr95, Tyr188, His249<br><br>D molecule: Asp63, Tyr95, Tyr188, His249	Carbonate ion			
6D04	Cryo-EM structure of a Plasmodium vivax invasion complex essential for entry into human reticulocytes; two molecules of parasite ligand, subclass 1	3.74	Fe^{3+}	Tyr426, Tyr517, His585	Carbonate ion	Fe_NFe_C-hTF	17	Cryo-EM structure containing 2 molecules of human transferrin in complex with transferrin receptor protein and reticulocyte binding protein
			Fe^{3+}	Asp63, Tyr95, Tyr188, His249	Carbonate ion			
6D05	Cryo-EM structure of a Plasmodium vivax invasion complex essential for entry into human reticulocytes; two molecules of parasite ligand, subclass 2	3.80	Fe^{3+}	Tyr426, Tyr517, His585, Arg632	Carbonate ion	Fe_NFe_C-hTF		Cryo-EM structure containing 2 molecules of human transferrin in complex with transferrin receptor protein and reticulocyte binding protein
			Fe^{3+}	Asp63, Tyr95, Tyr188, His249	Carbonate ion			
6UJ6	X-ray Crystal Structure of Chromium-transferrin with	2.68	Cr^{3+}	Asp392, Tyr426, Tyr517, His585	Malonate ion	Cr_C-hTF	18	Unusual sequence numbering (metal interacting residues: Asp411, Tyr445, Tyr536, His604)

<table>
<tr><td></td><td>Synergistic Anion Malonate</td><td></td><td></td><td></td><td></td><td></td><td></td><td></td></tr>
<tr><td>7Q1L</td><td>Glycosylated Human Serum Apo-transferrin</td><td>3.00</td><td>-</td><td>-</td><td>-</td><td>Apo-hTF</td><td>19</td><td>2 molecules in the asymmetric unit</td></tr>
<tr><td rowspan="2">5WTD</td><td rowspan="2">Structure of human serum transferrin bound ruthenium at N-lobe</td><td rowspan="2">2.50</td><td>Fe^{3+}</td><td>Asp392, Tyr426, Tyr517, His585</td><td>Malonate ion</td><td rowspan="2">Fe_C-hTF</td><td rowspan="15">2</td><td rowspan="2">The Ru^{3+} ion is bound to two residues of the N-lobe different from those of the Fe biding site</td></tr>
<tr><td>Ru^{3+}</td><td>His14, His289</td><td>Water</td></tr>
<tr><td rowspan="5">5X5P</td><td rowspan="5">Human serum transferrin bound to ruthenium NTA</td><td rowspan="5">2.70</td><td>Fe^{3+}</td><td>Asp392, Tyr426, Tyr517, His585</td><td>Malonate ion</td><td rowspan="5">Fe_C-hTF</td><td rowspan="5">The Ru^{3+} ions are bound to residues of the C-lobe (His578) or N-lobe (His14, His273, His289) different from those of the Fe biding sites. di-Ru^{3+} nuclear formation between two symmetric His273 created by crystal packing.</td></tr>
<tr><td>Ru^{3+}</td><td>His14</td><td>Nitrilotriacetic acid, water</td></tr>
<tr><td>Ru^{3+}</td><td>His578</td><td>Nitrilotriacetic acid</td></tr>
<tr><td>Ru^{3+}</td><td>His273</td><td>Ruthenium, Water</td></tr>
<tr><td>Ru^{3+}</td><td>His289</td><td>-</td></tr>
<tr><td rowspan="6">7FFM</td><td rowspan="6">Human serum transferrin with five osmium binding sites</td><td rowspan="6">3.06</td><td>Ti^{4+}</td><td>Asp392, Tyr426, Tyr517, His585</td><td>Malonate ion</td><td rowspan="6">Ti_C-hTF</td><td rowspan="6">The Os^{3+} ions are bound to residues of the C-lobe (His349, His350, Lys489, Lys490, Glu507, His578, Arg581) or N-lobe (His14, His289) different from those of the Fe biding sites</td></tr>
<tr><td>Os^{3+}</td><td>His14, His289</td><td>Nitrilotriacetic acid, water</td></tr>
<tr><td>Os^{3+}</td><td>Lys490, Glu507</td><td>Water</td></tr>
<tr><td>Os^{3+}</td><td>Lys489</td><td>Water</td></tr>
<tr><td>Os^{3+}</td><td>His578, Arg581</td><td>Water</td></tr>
<tr><td>Os^{3+}</td><td>His349, His350</td><td>Water</td></tr>
<tr><td rowspan="2">7FFU</td><td rowspan="2">Osmium-bound human serum transferrin</td><td rowspan="2">2.60</td><td>Fe^{3+}</td><td>Asp392, Tyr426, Tyr517, His585</td><td>Malonate ion</td><td rowspan="2">Fe_C-hTF</td><td rowspan="2">The Os^{3+} ion is bound to two residues of the N-lobe different from those of the Fe biding site</td></tr>
<tr><td>Os^{3+}</td><td>His14, His289</td><td>Water</td></tr>
<tr><td rowspan="2">6JAS</td><td rowspan="2">Human serum transferrin with iron citrate bound</td><td rowspan="2">2.50</td><td>Fe^{3+}</td><td>Asp392, Tyr426, Tyr517, His585</td><td>Malonate ion</td><td rowspan="2">Fe_C-hTF</td><td rowspan="2">M. Wang, H. Wang and H. Sun, to be published.</td><td rowspan="2">The second Fe^{3+} ion is close to N-lobe, but it does not interact with protein</td></tr>
<tr><td>Fe^{3+}</td><td>-</td><td>Citric acid, water</td></tr>
<tr><td colspan="9">[a] Apo-hTF = human transferrin with C- and N-lobes adopting the open conformation; M_C-hTF = human transferrin with the metal ion bound to C-lobe adopting the closed conformation; M_NM_C-hTF = human transferrin with metal ions bound to C- and N-lobes adopting the closed conformation.</td></tr>
</table>

Supplemental figures

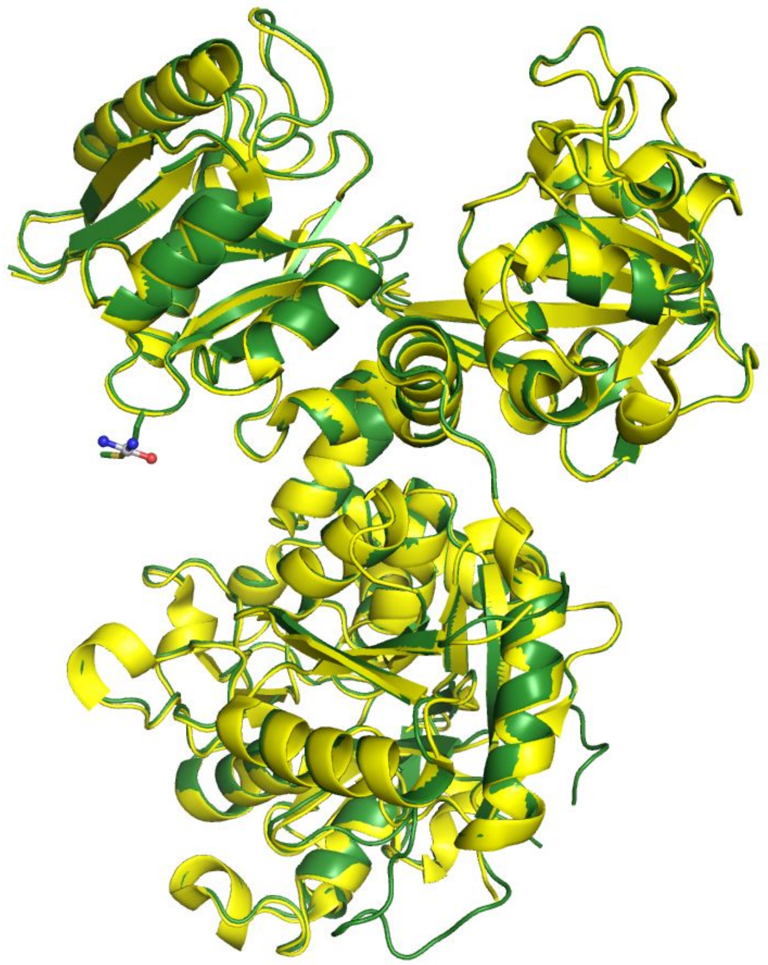

Figure S1. Superimposition of the cisplatin/Fe_C-hTF adduct (green) with the Pt-free Fe_C-hTF structure (yellow, PDB code: 4X1B) used as the search model to solve the phase problem.

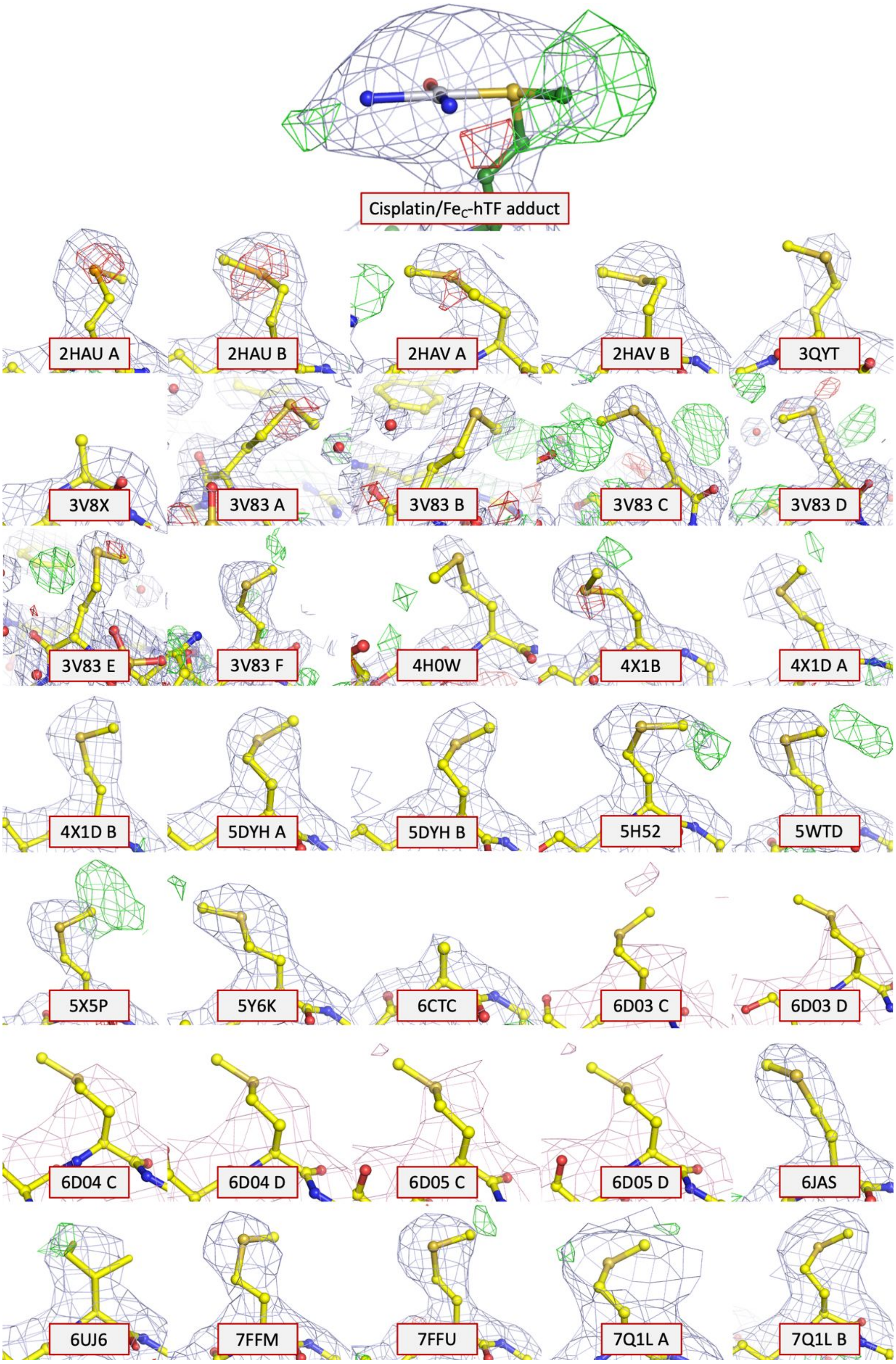

Figure S2. 2F$_o$-F$_c$ (grey, 1.0 σ value) and F$_o$-F$_c$ (green/red, 3.0 σ value) electron density maps and cryo-EM maps (light pink) close to Met256 in the cisplatin/Fe$_C$-hTF adduct here solved (on the top) and in the other structures of hTF deposited in the Protein Data Bank. If more hTF molecules are in the model, the chain names are indicated after the PDB code. Se-Met and Ile residues are present in place of Met in 2HAU and 6UJ6 structures, respectively. The atoms of the Met side chain other than C_α are missing in 3V8X and 6CTC models.

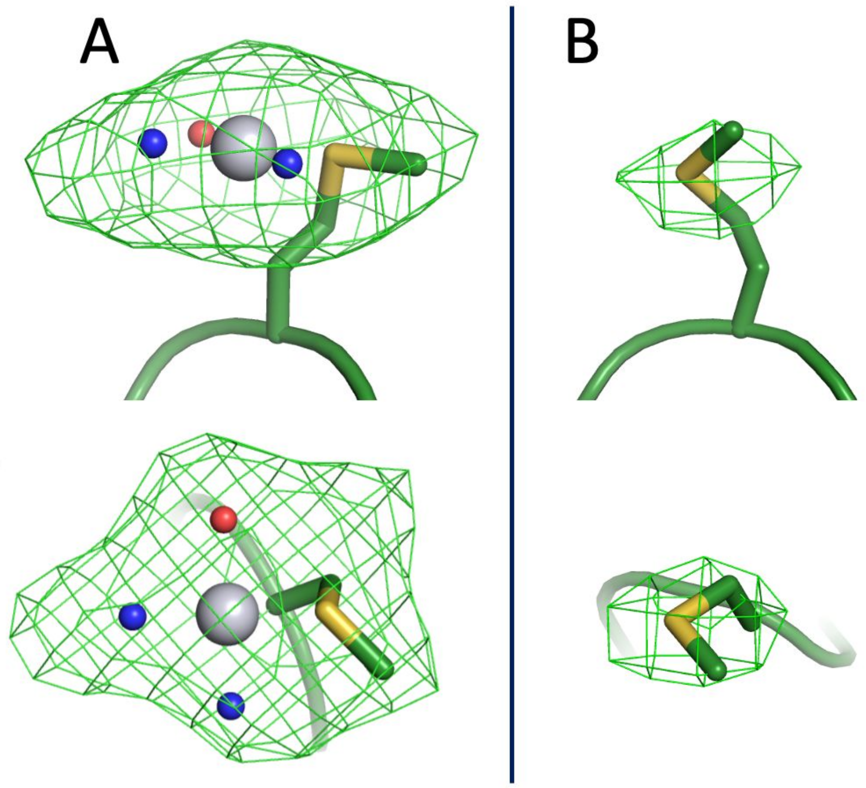

Figure S3. Omit F_o-F_c electron density maps in correspondence of the side chain of Met256 in the structures of cisplatin/Fe_C-hTF adduct (A, 3.0 σ value) and the Pt-free Fe_C-hTF (B, 2.0 σ value).

References

(1) Wang, M.; Lai, T. P.; Wang, L.; Zhang, H.; Yang, N.; Sadler, P. J.; Sun, H. "Anion Clamp" Allows Flexible Protein to Impose Coordination Geometry on Metal Ions. *Chem Commun (Camb)* **2015**, *51* (37), 7867–7870. https://doi.org/10.1039/c4cc09642h.

(2) Wang, M.; Wang, H.; Xu, X.; Lai, T.-P.; Zhou, Y.; Hao, Q.; Li, H.; Sun, H. Binding of Ruthenium and Osmium at Non-iron Sites of Transferrin Accounts for Their Iron-Independent Cellular Uptake. *J Inorg Biochem* **2022**, *234*, 111885. https://doi.org/10.1016/j.jinorgbio.2022.111885.

(3) Evans, P. Scaling and Assessment of Data Quality. *Acta Cryst D* **2006**, *62* (Pt 1), 72–82. https://doi.org/10.1107/S0907444905036693.

(4) Kabsch, W. XDS. *Acta Cryst D* **2010**, *66* (Pt 2), 125–132. https://doi.org/10.1107/S0907444909047337.

(5) Winn, M. D.; Ballard, C. C.; Cowtan, K. D.; Dodson, E. J.; Emsley, P.; Evans, P. R.; Keegan, R. M.; Krissinel, E. B.; Leslie, A. G. W.; McCoy, A.; McNicholas, S. J.; Murshudov, G. N.; Pannu, N. S.; Potterton, E. A.; Powell, H. R.; Read, R. J.; Vagin, A.; Wilson, K. S. Overview of the CCP4 Suite and Current Developments. *Acta Cryst D* **2011**, *67* (Pt 4), 235–242. https://doi.org/10.1107/S0907444910045749.

(6) Vonrhein, C.; Flensburg, C.; Keller, P.; Sharff, A.; Smart, O.; Paciorek, W.; Womack, T.; Bricogne, G. Data Processing and Analysis with the AutoPROC Toolbox. *Acta Cryst D* **2011**, *67* (Pt 4), 293–302. https://doi.org/10.1107/S0907444911007773.

(7) McCoy, A. J.; Grosse-Kunstleve, R. W.; Adams, P. D.; Winn, M. D.; Storoni, L. C.; Read, R. J. Phaser Crystallographic Software. *J Appl Crystallogr* **2007**, *40* (Pt 4), 658–674. https://doi.org/10.1107/S0021889807021206.

(8) Murshudov, G. N.; Skubák, P.; Lebedev, A. A.; Pannu, N. S.; Steiner, R. A.; Nicholls, R. A.; Winn, M. D.; Long, F.; Vagin, A. A. REFMAC5 for the Refinement of Macromolecular Crystal Structures. *Acta Cryst D* **2011**, *67* (Pt 4), 355–367. https://doi.org/10.1107/S0907444911001314.

(9) Emsley, P.; Lohkamp, B.; Scott. W. G.; Cowtan, K. Features and Development of Coot. *Acta Cryst D* **2010**, *66* (Pt 4), 486–501. https://doi.org/10.1107/S0907444910007493.

(10) Wally, J.; Halbrooks, P. J.; Vonrhein, C.; Rould, M. A.; Everse, S. J.; Mason, A. B.; Buchanan, S. K. The Crystal Structure of Iron-Free Human Serum Transferrin Provides Insight into Inter-Lobe Communication and Receptor Binding. *J Biol Chem* **2006**, *281* (34), 24934–24944. https://doi.org/10.1074/jbc.M604592200.

(11) Yang, N.; Zhang, H.; Wang, M.; Hao, Q.; Sun, H. Iron and Bismuth Bound Human Serum Transferrin Reveals a Partially-Opened Conformation in the N-Lobe. *Sci Rep* **2012**, *2*, 999. https://doi.org/10.1038/srep00999.

(12) Noinaj, N.; Easley, N. C.; Oke, M.; Mizuno, N.; Gumbart, J.; Boura, E.; Steere, A. N.; Zak, O.; Aisen, P.; Tajkhorshid, E.; Evans, R. W.; Gorringe, A. R.; Mason, A. B.; Steven, A. C.; Buchanan, S. K. Structural Basis for Iron Piracy by Pathogenic Neisseria. *Nature* **2012**, *483* (7387), 53–58. https://doi.org/10.1038/nature10823.

(13) Tinoco, A. D.; Saxena, M.; Sharma, S.; Noinaj, N.; Delgado, Y.; Quiñones González, E. P.; Conklin, S. E.; Zambrana, N.; Loza-Rosas, S. A.; Parks, T. B. Unusual Synergism of Transferrin and Citrate in the Regulation of Ti(IV) Speciation, Transport, and Toxicity. *J Am Chem Soc* **2016**, *138* (17), 5659–5665. https://doi.org/10.1021/jacs.6b01966.

(14) Curtin, J. P.; Wang, M.; Cheng, T.; Jin, L.; Sun, H. The Role of Citrate, Lactate and Transferrin in Determining Titanium Release from Surgical Devices into Human Serum. *J Biol Inorg Chem* **2018**, *23* (3), 471–480. https://doi.org/10.1007/s00775-018-1557-5.

(15) Jiang, N.; Cheng, T.; Wang, M.; Chan, G. C.-F.; Jin, L.; Li, H.; Sun, H. Tracking Iron-Associated Proteomes in Pathogens by a Fluorescence Approach. *Metallomics* **2018**, *10* (1), 77–82. https://doi.org/10.1039/c7mt00275k.

(16) Pratt, R.; Handelman, G. J.; Edwards, T. E.; Gupta, A. Ferric Pyrophosphate Citrate: Interactions with Transferrin. *Biometals* **2018**, *31* (6), 1081–1089. https://doi.org/10.1007/s10534-018-0142-2.

(17) Gruszczyk, J.; Huang, R. K.; Chan, L.-J.; Menant, S.; Hong, C.; Murphy, J. M.; Mok, Y.-F.; Griffin, M. D. W.; Pearson, R. D.; Wong, W.; Cowman, A. F.; Yu, Z.; Tham, W.-H. Cryo-EM Structure of an Essential Plasmodium Vivax Invasion Complex. *Nature* **2018**, *559* (7712), 135–139. https://doi.org/10.1038/s41586-018-0249-1.

(18) Petersen, C. M.; Edwards, K. C.; Gilbert, N. C.; Vincent, J. B.; Thompson, M. K. X-Ray Structure of Chromium(III)-Containing Transferrin: First Structure of a Physiological Cr(III)-Binding Protein. *J Inorg Biochem* **2020**, *210*, 111101. https://doi.org/10.1016/j.jinorgbio.2020.111101.

(19) Campos-Escamilla, C.; Siliqi, D.; Gonzalez-Ramirez, L. A.; Lopez-Sanchez, C.; Gavira, J. A.; Moreno, A. X-Ray Characterization of Conformational Changes of Human Apo- and Holo-Transferrin. *Int J Mol Sci* **2021**, *22* (24), 13392. https://doi.org/10.3390/ijms222413392.
